# A reciprocal feedback of miR-548ac/YB-1/Snail induces EndMT of HUVECs during acidity microenvironment

**DOI:** 10.1186/s12935-021-02388-8

**Published:** 2021-12-20

**Authors:** Jingyuan Chen, Shengbo Han, Jinhuang Chen, Ping Hu, Zhu Zeng, Yuhang Hu, Hewei Xiong, Zunxiang Ke, Ya Zhang, Fengyu Xu, Gang Zhao

**Affiliations:** Department of Emergency Surgery, Union Hospital, Tongji Medical College, Huazhong University of Science and Technology, Wuhan, 430022 China

**Keywords:** EndMT, Pancreatic cancer, Transmigration, Acidity, miR-548ac, Snail

## Abstract

**Background:**

Researches indicated the process of Endothelial-Mesenchymal-Transition (EndMT) of vascular endothelial cells (ECs) was critically involved in the progression of tumor. ECs demonstrated functional and phenotypic heterogeneity when located under different microenvironments. The extracellular pH of tumor tissues was acidic compared to that of normal tissues. However, there was still unclear whether the acidic microenvironment affected the EndMT of vascular ECs.

**Methods:**

Human Umbilical Vein Endothelial Cell (HUVECs) was cultured under the normal or acidic medium to evaluate the alteration of morphology, migration, permeability, and EndMT markers. Microarray assay was adopted to analyze the differential expression of miRNAs in the acidity-treated HUVECs. Gain- and loss- of function experiments were performed to evaluate the functional role of miRNA-548ac on acidity-induced EndMT of HUVECs. Luciferase reporter and Chromatin-immunoprecipitation assays were conducted to assess the downstream pathway of miRNA-548ac in acidity-induced EndMT of HUVECs.

**Results:**

Our results showed that HUVECs demonstrated mesenchymal transition under acidic conditions with the increase of migration, permeability, and expression of α-SMA and Vimentin, but the expression of vascular endothelial cadherin (VE-cadherin) and CD31 were reduced. In addition, the acidity-treated HUVECs remarkably facilitated the transmigration of pancreatic cancer cells. The expression of miRNA-548ac was significantly decreased in the acidity-treated HUVECs. Moreover, overexpression of miR-548ac inhibited the EndMT of HUVECs and consequently impeded the transmigration of pancreatic cancer cells. The miR-548ac inhibited the expression of YB-1 by binding to the 3’UTR of its mRNA, and YB-1 promoted the translation of Snail which was a critical regulator of EndMT. What’s more, Snail transcriptionally inhibited the expression of miR-548ac through binding to the promoter of its host gene.

**Conclusions:**

Our data implicated that the acidic microenvironment promoted the EndMT of HUVECs by the miR-548ac/YB-1/Snail axis, which could contribute to the metastasis of pancreatic cancer.

**Supplementary Information:**

The online version contains supplementary material available at 10.1186/s12935-021-02388-8.

## Background

Pancreatic cancers often appear metastases in the early phase of the disease, the malignant progression of pancreatic cancer resulting in a poor prognosis for patients with a 5-year survival rate of <5%. During the process of cancer metastasis, the metastasizing cancer cells need to weaken the interendothelial junctions to cross the endothelial barrier [1–3]. Research has highlighted the critical role of the breakdown of impermeable vasculature endothelial and it is believed to be one of the major rate-limiting steps in cancer metastasis [4, 5].

Endothelial-to-mesenchymal transition (EndMT) is a process that resident endothelial cells (ECs) delaminate from an organized cell layer and acquire a mesenchymal phenotype characterized by loss of cell-cell junctions, loss of endothelial markers, gain mesenchymal markers, increase in permeability, and acquisition of invasive and migratory properties. EndMT has been proved to be a vital biological process in heart development [6]. Recent evidence suggested that EndMT could occur postnatally in a variety of pathologic settings, including cardiac fibrosis [7], chronic pulmonary hypertension [8], and cancer [9]. Considering cancer, most recent research described EndMT as a mechanism for the generation of Cancer-associated fibroblasts (CAFs) [7, 10]. However, the acquisition of the mesenchymal phenotype of endothelial cells (ECs) during EndMT and its significance in tumor propagation has never been explained earlier.

The extracellular pH (pHe) of tumor tissues is often acidic compared to that of normal tissues (6.2–6.9 vs. 7.3–7.4) [11–13], and acidic metabolites, e.g., lactic acid is caused by anaerobic glycolysis in hypoxia, appearing to be the main cause [14]. Accumulating evidence indicated that acidity of the tumor microenvironment promoted the local growth, invasion, and metastasis of tumors [15–17]. What’s more, evidence suggested that acidic pHe might act as a microenvironment stimulus in the process of cancer models inducing epithelial-to-mesenchymal transition (EMT) [18, 19]. In consistent with our previous study, acidity suppressed the expression of miR-642, which further promoted the EMT process and remarkably enhanced the invasive ability of pancreatic cancer cells [20].

Increasing research indicated that microRNAs (miRNAs), acting as the crucial stress-responsive mediator, were involved in the signal transduction in response to stress including hypoxia, inflammation, and acidity stimulus [20–23]. Recently, a wealth of evidence from our laboratory and others highlighted the role of miRNAs in the EMT process [20–22, 24–26]. However, the expression levels of miRNAs and their implication in EndMT of tumor ECs under acidic microenvironment were still unknown.

YB-1 (Y-box binding protein-1), a DNA/RNA-binding protein, was a member of the cold-shock domain protein superfamily and involved in fundamental processes such as DNA repair, mRNA transcription, translation, and stabilization [27]. Recently, numerous studies pointed out the role of YB-1 in tumor progression including pancreatic cancer [28]. In addition, Evdokimova et al. identified YB-1 as a new player in the regulation of EMT through a novel mechanism involving translation of cap-dependent mRNAs encoding Snail and other EMT regulators in Ras-transformed cells [27]. Since numerous studies have implicated Snail acted as a transcription factor in the regulation of various EMT during embryogenesis and cancer progression, Snail has been highlighted as a master regulator of cell plasticity [29–32]. Moreover, Snail has been regarded as a universal transcriptional repressor by directly targeting numerous genes involved in cellular transitions, through an E-box sequence with a consensus of six base pairs (CAGCTG) [33].

In our present study, we aimed to elucidate the potential role of acidity in the induction of EndMT involving endothelial cells and its novel mechanism, which might provide novel therapeutic targets for pancreatic cancer.

## Materials and methods

### Cell culture

The human umbilical vein vascular endothelial cells (HUVECs) and human pancreatic cancer cell lines (PANC-1, BxPC-3) were obtained from ATCC (South San Francisco, USA) and they were tested and authenticated for genotypes by DNA fingerprinting. These cell lines were passaged for <6 months after resuscitation. These cell lines were cultured in RPMI-1640 medium (HyClone, Logan, UT, USA) supplemented with 10% fetal bovine serum (HyClone), 100 U/mL penicillin, and 100 μg/mL streptomycin under a humidified 5% CO_2_, 95% air atmosphere at 37 ℃. To vary the medium pH, we added 20 mM 2-(N-morpholino)-ethane-sulfonic acid and 20 mM Tris-(hydroxymethyl)-aminomethane [34].

### Transfection of microRNA, siRNA, and plasmid

Inhibitors and mimics of miR-548ac, YB-1 siRNAs and the corresponding negative controls were obtained from RiboBio (Guangzhou, China). The Snail and YB-1 overexpression plasmid and the empty vector were obtained from Genechem (Shanghai, China). The lipofectamine 2000 reagent was obtained from Invitrogen (Invitrogen, Carlsbad, CA, USA) and then transfection steps of plasmid DNA, siRNAs, inhibitors, and mimics of miR-548ac were conducted according to the manual protocol.

### MiRNA array

The miRNA array was carried out by Sangon Biotech (Shanghai, China). Briefly, total RNAs were extracted from HUVECs cells on normal and acidic conditions for 48 hours using TRIzol reagent (Invitrogen, USA). Then, they were labeled with cyanine 3-pCp molecule at the 3′ end. After purification of labeled RNA, they were hybridized overnight with target microRNA. At last, the fluorescence was detected by GenePix 400B laser scanner (Molecular Device, USA) and the data was analyzed by Gene Spring GX software.

### Western blot assay and qRT-PCR

Protein extracts were separated by 10% polyacrylamide gels and transferred to PVDF membranes (Millipore). Blots were incubated with the following primary antibodies: VE-cadherin (Proteintech, 66804-1-Ig), CD31 (Proteintech, 11265-1-AP), α-SMA (Proteintech, 55135-1-AP), Vimentin (Proteintech, 60330-1-Ig), GAPDH (Proteintech, 10494-1-AP), YB1 (Proteintech, 20339-1-AP), Snail (Proteintech, 20339-1-AP) overnight at 4 °C. Then incubated with corresponding secondary antibodies. Image J (National Institutes of Health) calculated the band density.

Total RNA extraction was collected by RNAiso Plus kit (TAKARA) according to the protocol. DNA was synthesized by the PrimeScript^®^ RT Master Mix Perfect Real-Time kit (TAKARA) according to the protocol. SYBR Premix Ex Taq II (TAKARA) was used to perform PCR amplification. The expression of VE-cadherin, CD31, α-SMA, Vimentin, YB-1, Snail were normalized by GAPDH. Primer sequences were all shown in Additional file 5: Table S1.

### Endothelial permeability assay

Permeability across endothelial cell monolayers was measured by using Boyden chambers for 24-well plates (0.8-µm pore size; Corning, Fisher Scientific) coated with ECM gel (40 μL, Sigma) according to the manufacturer’s instructions as performed previously [35]. Suspensions of 5 × 10^4^ transfected or non-transfected HUVECs in 100 µL medium were added to the upper chambers and incubated with normal (pH 7.4) or acidic medium (pH 7.0; pH 6.8) for 24–48 h until the formation of a tight monolayer. Thereafter, Rhodamine-labelled dextran (Dextran-Rhodamine, 1 mg/mL, MW: 70 kDa) was plated into the upper chambers. Endothelial permeability was determined by measuring the passage of Rhodamine-labelled dextran across HUVECs monolayers to the bottom chambers at 0, 10, 30, and 60 min [35].

### Transendothelial cell migration assay

Pancreatic cancer cell migration through the endothelial barrier was performed in Boyden chambers. HUVECs were seeded onto the upper compartment of Boyden chambers as described above (see “Endothelial permeability assay”). Thereafter, both normal and acidic mediums were removed and replaced by a fresh medium (pH7.4), and the GFP labeled pancreatic cancer cell lines (BxPC-3-GFP with 2 × 10^4^ and PANC-1-GFP with 6 × 10^4^) were placed onto the surface of the upper compartment. After another 24 h, the pancreatic cancer cells that had penetrated the filter into the bottom of the compartment were counted in five different fields using cell images that were visualized using a scanning confocal fluorescence microscope (Olympus Co., Japan) at ×100 magnification [21].

### Cell migration assay

Migration assays were performed in triplicate using Transwell chambers for 24-well plates (0.8-µm pore size; Corning, Costar) coated with ECM gel (40 µL; Sigma) according to the manufacturer’s instructions. BxPC-3 cells (2 × 10^4^) and PANC-1 cells (6 × 10^4^) were plated in 200 µL RPMI 1640 medium with 0.1% fetal bovine serum into the upper chamber. The lower chamber was filled with 700 µL RPMI 1640 medium with 30% fetal bovine serum. After culturing for 24–72 h, noninvaded cells were mechanically removed by a cotton swab. The invaded cells on the underside of the membrane were fixed with 4% formalin and stained with 0.1% crystal violet for visualization. Cells were counted in ten respective microscopic fields (×40 magnification) and photographed.

### Wound-healing assay

For the wound-healing assay, the cells were seeded in 12-well plates and allowed to grow until their concentration reached 90%. The cell monolayer was scratched by an artificial “wound” carefully created at 0 h, using a 10 μL pipette tip on the confluent cell monolayer, microphotographs were taken at 0 and 48 h (×20 magnification).

### Chromatin Immunoprecipitation analysis (ChIP)

For Chromatin Immunoprecipitation analysis (ChIP), we obtained the EZ ChIP kit from Merck Millipore (Catalog #17-371). The procedures were conducted as follows: Firstly, the cells to be tested were cross-linking by formaldehyde, then cells were collected and treated with SDS lysis buffer. Secondly, cell lysate was exposed to sonication on wet ice, shearing the crosslinked DNA to 200–1000 bp in length. Thirdly, non-specifically bound impurities were removed by Protein G agarose, and then 1–10 μg of antibody (anti-Snail or IgG) was added into each sample to be tested, incubated overnight at 4 °C with rotation, and then protein G Agarose was incubated with the sample to collect the antibody/protein/DNA complex. Further, Protein/DNA Complexes were eluted from protein G Agarose, and then free DNA was acquired from Protein/DNA Complexes by reverse crosslinks. Lastly, the free DNA fragments were purified and detected binding sites of interest by PCR.

### Luciferase assay

We constructed four luciferase reporter plasmids containing the miR-548ac host-gene promoter sequences with wild type, mutant-1, mutant-2, or mutant-3 E-box sites. HUVECs were co-transfected with Snail overexpression plasmid or the negative control vector and the WT or MUT luciferase reporter plasmid along with the Renilla luciferase vector as an internal control. Then the luciferase activity was measured by the Dual-Luciferase^®^ Reporter Assay System from Promega (Catalog #E1910).

### Statistical analysis

The statistical analysis was conducted by SPSS 17.0 (IBM, USA). The student’s t test was used to assess differences between groups. All data were presented as mean ± SEM. Statistical significance was showed as “*p <0.05, **p <0.01, ***p <0.001, and ns means no significant.

## Results

### Acidity induces EndMT of HUVECs

**Fig. 1:**
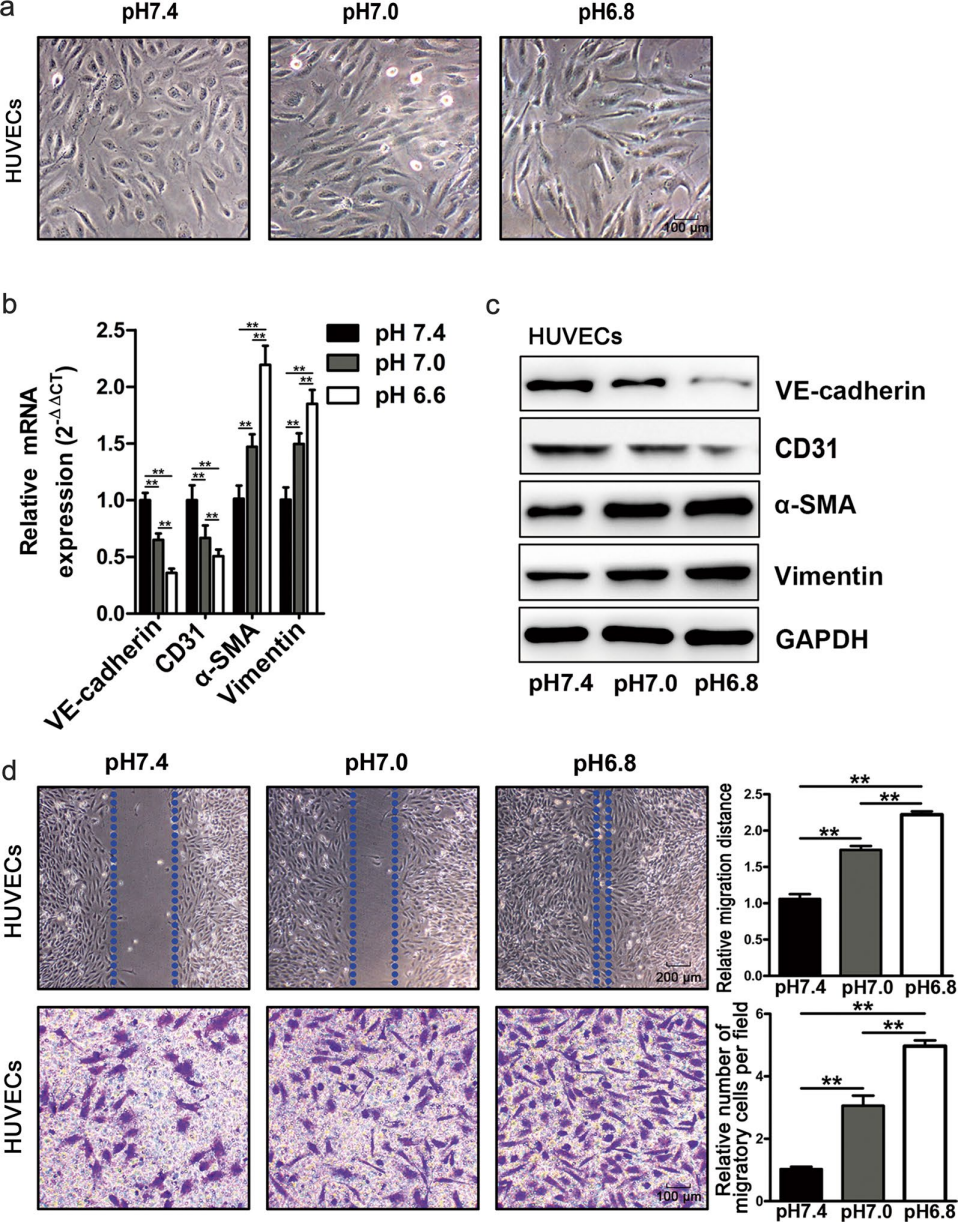
Acidity induces the endothelial-to-mesenchymal transition of HUVECs. **a** The morphology of HUVECs in the normal and conditioned medium (pH 7.0 pH 6.8). **b**, **c** The expression of vascular endothelial markers including VE-cadherin, CD31, α-SMA, and Vimentin at mRNA and protein levels in normal and conditioned medium (pH 7.0 pH 6.8). **d** The wound-healing and transwell migration assay of HUVECs in normal and conditioned medium (pH 7.0 pH 6.8), the histogram is the statistical result of the corresponding wound-healing and transwell migration assay

To explore the effect of acidic pH_e_ on endothelial cells (ECs), HUVECs were incubated in normal (pH 7.4) or different acidic mediums (pH 7.0, pH 6.8). Upon incubation in the acidic medium for 48 h, the HUVECs underwent a morphological change, from epithelioid to elongated and spindle-shaped fibroblast-like appearance (Fig. 1a). In addition, the acidity-treated HUVECs took on the reduction of vascular endothelial markers including vascular endothelial cadherin (VE-cadherin) and CD31, as well as an increase of α-SMA and Vimentin both at mRNA and protein levels (Fig. 1b, c; Additional file 1: Fig. S1e). Moreover, the wound-healing assay and transwell assay showed that HUVECs became more dynamic in a pH-dependent manner (Fig. 1d) after incubation in the acidic medium (pH7.0, pH 6.8).

### Acidity-induced EndMT promotes the permeability of HUVECs

**Fig. 2:**
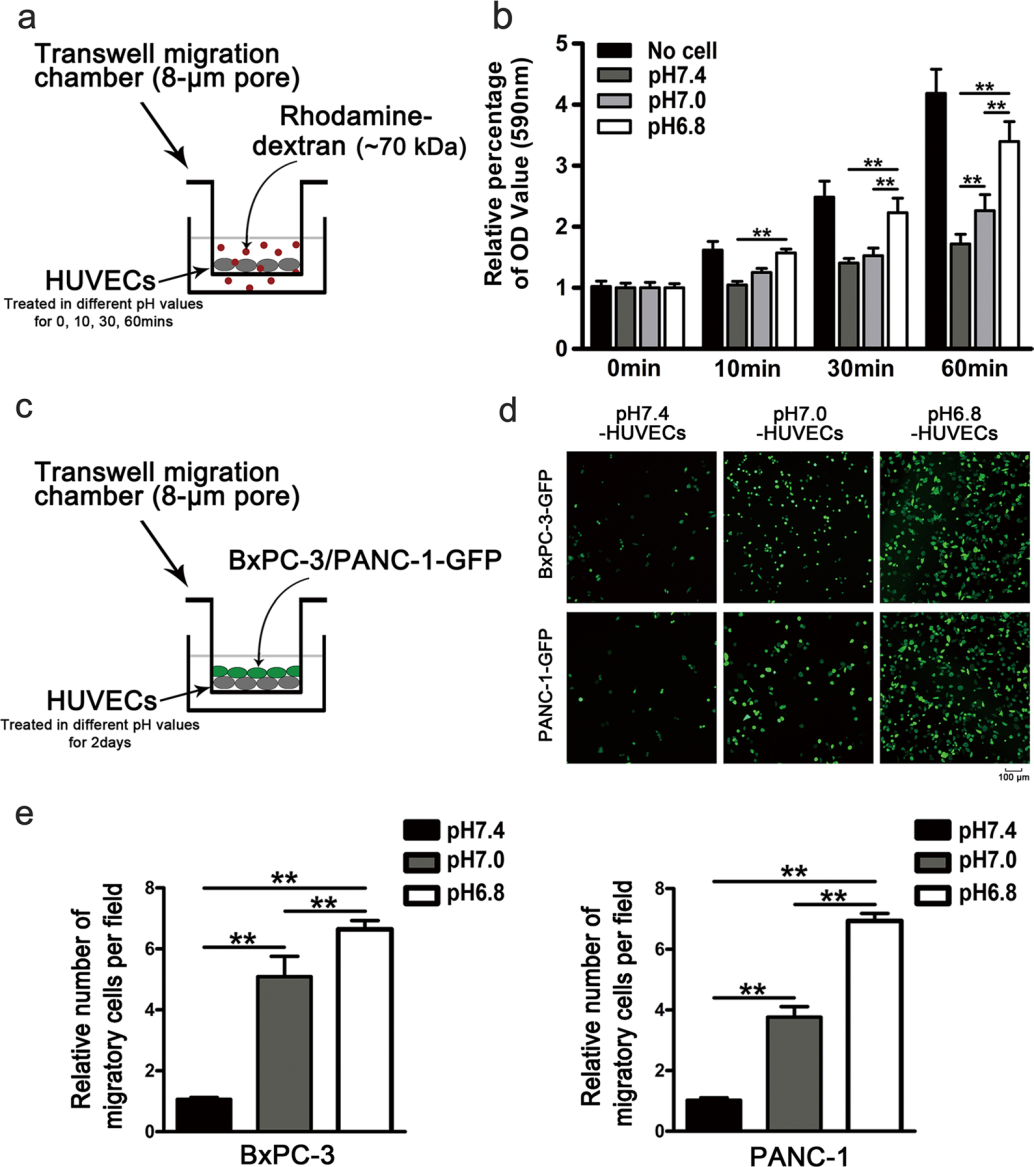
Acidity induces endothelial cell permeability. **a**, **b** The endothelial permeability was determined by measuring the passage of Rhodamine-labelled dextran across HUVECs monolayers to the bottom chambers at 0, 10, 30, and 60 min, respectively. **c**–**e** The transendothelial migration assay of BxPC-3 and PANC-1 cells migrated into chambers with monolayer HUVECs treated with the acidic medium. The histogram represents the relative number of BxPC-3 and PANC-1 cells that crossed the HUVECs monolayers

Studies had addressed the breakdown of VE-cadherin during EndMT led to altered vascular permeability and remolding, which were associated with many disease processes including angiogenesis, ischemia-reperfusion injury, inflammation, and cancer growth and metastasis [36, 37]. Accordingly, we further ascertained whether acidity induced an increase in the permeability of endothelial cell. HUVECs were cultivated as tight monolayers in Boyden chambers and treated with normal (pH7.4) or acidic medium (pH7.0; pH6.8) for 0, 10, 30, and 60 min. Thereafter, permeability was determined by measuring the passage of Rhodamine-labelled dextran (Dextran-Rhodamine, MW: 70 kDa) across endothelial cell monolayers (Fig. 2a). Results showed that acidity remarkably promoted the passage of the Rhodamine-labelled dextran from the top to the bottom in a time and pH-dependent manner, which indicated an increased permeability of endothelial cells (Fig. 2b).

### Acidity-induced EndMT in HUVECs facilitates transmigration of pancreatic cancer cells

Since EndMT was linked to a reduced capacity of the vasculature which averted tumor cell intra- and extravasation, we have further evaluated whether the acidity-induced EndMT of ECs enhanced transmigration of pancreatic cancer cells. The transendothelial migration assay was shown in Fig. 2c, HUVECs were seeded onto the upper compartment of transwell chambers and incubated in normal (pH7.4) or acidic medium (pH7.0; pH6.8) for 2 days. After that, both normal and acidic mediums were removed and replaced by a fresh medium (pH7.4), and the pancreatic cancer cell lines (BxPC-3-GFP and PANC-1-GFP) were placed onto the surface of the upper compartment. After another 24 h, the pancreatic cancer cells that had penetrated the filter into the bottom of the compartment were counted. The results showed that when co-cultured with HUVECs treated with acidic medium, more BxPC-3 and PANC-1 cells migrated into the below compartment (Fig. 2d, e). Together, these results indicated that the acidic-induced EndMT of ECs facilitated the transendothelial migration of pancreatic cancer cells by enhancing the permeability of the endothelial barrier.

### The downregulation of miRNA-548ac is critical for acidity-induced EndMT in HUVECs

**Fig. 3:**
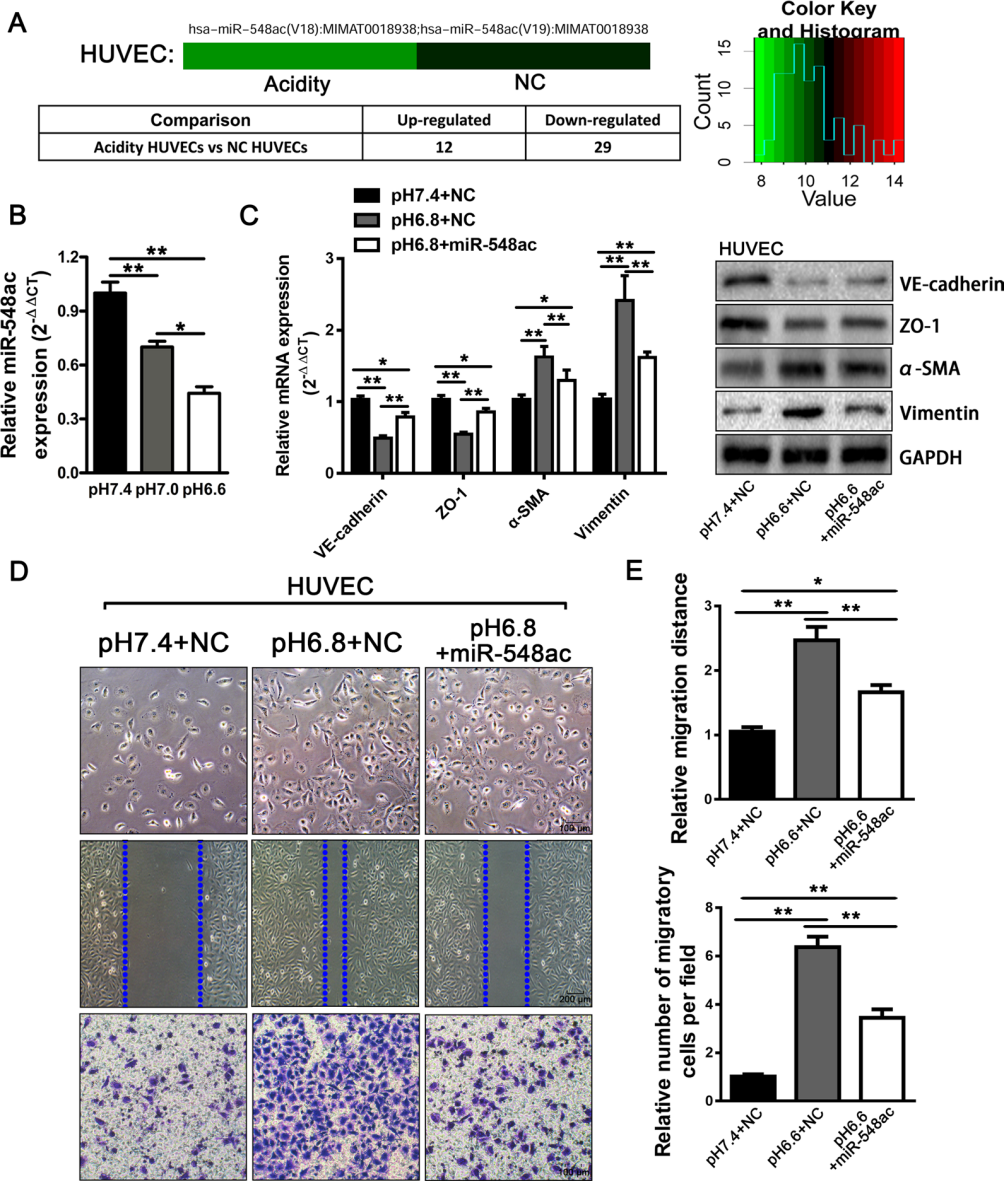
Downregulation of miRNA-548ac under acidity is critical for EndMT in HUVECs. **a** The miRNA array data screened the dysregulated miRNAs varied more than 1.5-folds in the HUVECs treated with acidic medium (pH 6.8). **b** The relative expression of miR-548ac in acidic medium (pH 7.0, pH 6.8) compared to normal (pH 7.4). **c** The expression of vascular endothelial markers including VE-cadherin, CD31, α-SMA, and Vimentin at mRNA and protein levels in HUVECs in normal, acidic medium or acidic medium with miR-548ac mimics. **d** The morphology, wound-healing, and transwell migration assay of HUVECs when cells were cultured in normal, acidic medium or acidic medium with miR-548ac mimics. Histograms are the statistical result of the corresponding wound-healing and transwell migration assay

Accumulative researches had shown that miRNAs could act as a crucial stress-responsive mediator, which were involved in signal transduction in response to stress including hypoxia, inflammation, and acidic stimulus [20–23]. Based on these findings, we performed a miRNA array on the total RNA isolated from HUVECs (pH 7.4 vs. pH 6.8) to figure out miRNAs that might contribute to the acidity-induced EndMT of HUVECs. The miRNA array data revealed that there were 12 miRNAs upregulated and 29 miRNAs downregulated more than 1.5-folds in the HUVECs treated with the acidic medium (pH 6.8) (Fig. 3a). Among those decreased miRNAs, miRNA-548ac showed the most significant alteration. We further used qRT-PCR to detect the expression of miR-548ac in HUVECs after exposure to the acidic microenvironment. The result showed the expression of miR-548ac in HUVECs was decreased according to the descent of the pH value (Fig. 3b). To further evaluate the effects of miR-548ac on the acidity-induced EndMT, acidity-treated HUVECs (pH 6.8) were transfected with miR-548ac mimics or mimics control. After the restoration of miR-548ac, the acidity-treated HUVECs (pH 6.8) demonstrated a dramatic mesenchymal-to-endothelial transition like transformation with increased expression of VE-cadherin and CD31 and decreased expression of α-SMA and Vimentin (Fig. 3c; Additional file 1: Fig. S1f). Furthermore, the restoration of miR-548ac also remarkably attenuated the motility of HUVECs treated with acidity (pH 6.8), which were analyzed by wound-healing and transwell migration assay (Fig. 3d).

Consistently, the permeability of HUVECs under acidic conditions was dramatically repressed in the miR-548ac mimics group compared to the mimics control group (Additional file 1: Fig. S1a). Furthermore, our results also revealed that the restoration of miR-548ac significantly receded the transendothelial migration of pancreatic cancer cells (BxPC-3 and PANC-1) across the HUVECs barrier under acidic conditions (Additional file 1: Fig. S1b, c). What’s more, our data showed that the viability of HUVECs had no significant difference on the pH7.4, pH7.0, or pH6.8 conditions (Additional file 1: Fig. S1d). All these findings implied that the aberrantly expressed miR-548ac was essential for the acidity-induced EndMT of HUVECs.

### YB-1 is the direct target of acidity-inhibited miR-548ac of HUVECs

**Fig. 4:**
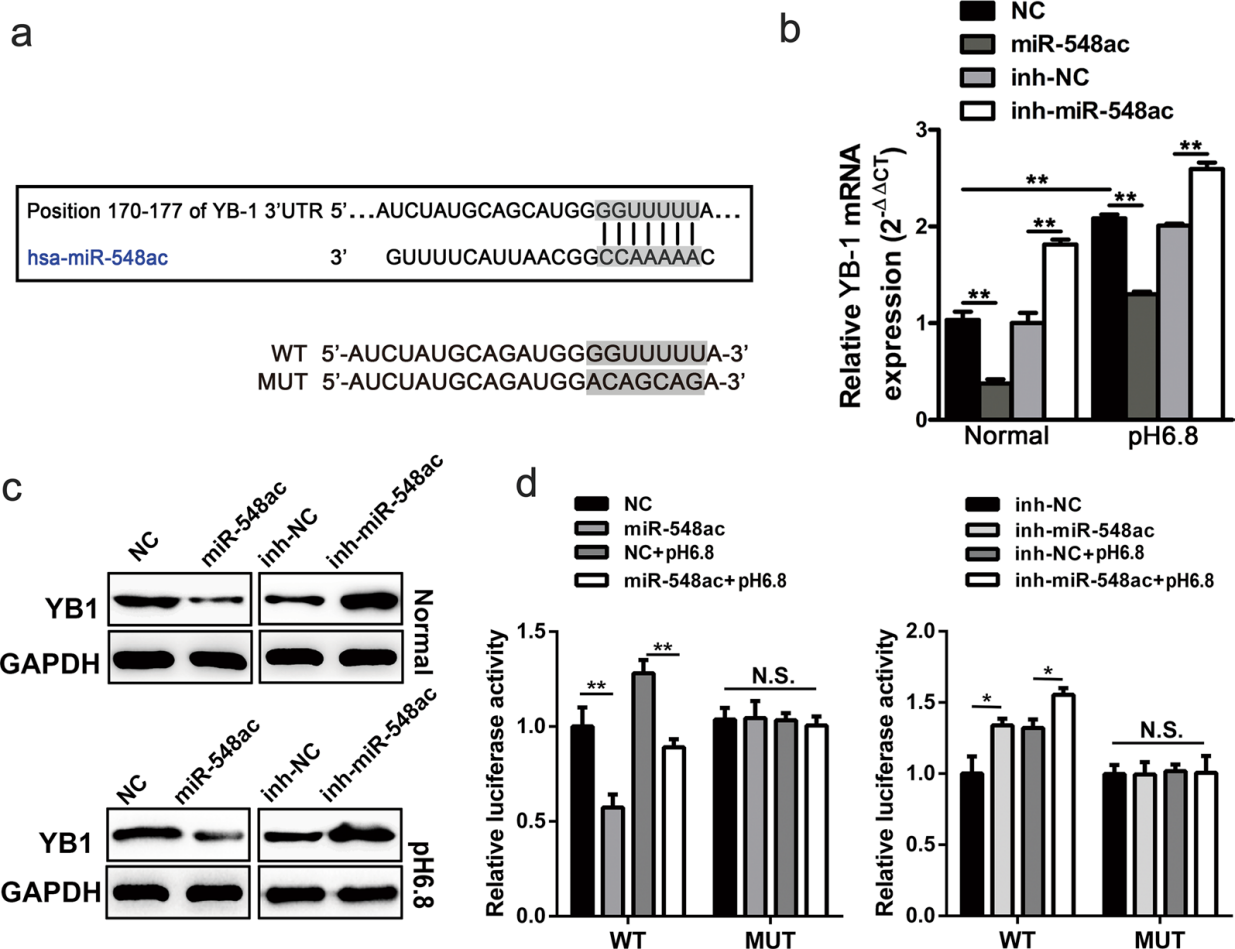
YB-1 is the direct target of miR-548ac. **a** Bioinformatic algorithms predicted miR-548ac might interact 3′UTR of YB-1 at the position of 170–177 and 262–269 nucleotides, the mutant sequences of MUT Vector mentioned below are shown as indicated. **b**, **c** After inhibition or overexpression of miR-548ac in the normal or acidic medium, the expression of YB-1 mRNA and protein were measured by qPCR and western blot, respectively. **d** The pMIR-REPORT luciferase vector containing 3′UTR of YB-1 sequences: the wild type of miR-548ac target sequence (WT) or the corresponding mutant type sequence (MUT) were co-transfected with inhibitors or mimics of miR-548ac in normal or acidic medium. Then the activity of luciferase density was measured

As miR-548ac shown to be a suppressor in the regulation of EndMT in HUVECs, we then screened the top 100 molecules which might be biological targets of miR-548ac by the database. YB-1 attracted our attention since the regulation of YB-1 was mediated by miRNAs in a wide variety of cancers [38, 39] and YB-1 was involved in tumor angiogenesis [40]. Importantly, bioinformatic algorithms predicted that miR-548ac might target the 3′UTR of YB-1 (position 170–177 and position 262–269) (Fig. 4a). Therefore, we speculated YB-1 might be controlled by miR-548ac under acidic conditions. Coincidently, the results showed overexpression of miR-548ac led to a decrease in the expression of YB-1, while knockdown of miR-548ac increased the expression of YB-1 in HUVECs both at mRNA and protein levels (Fig. 4b, c; Additional file 1: Fig. S1g). To further verify whether miR-548ac exerted its function by directly targeting 3′UTR of YB-1, we cloned 3′UTR of YB-1 sequences containing the target sequence (wild type, WT) or a mutated sequence (mutant type, MUT) into pMIR-REPORT luciferase vector, the mutant sequences of YB-1 3′UTR were also shown in Fig. 4a. The results showed that the activity of luciferase density in HUVECs with WT was considerably increased in acidity, which was reversed by co-transfection with miR-548ac mimics and enhanced by co-transfection with inh-miR548ac, but without alteration in HUVECs with MUT (Fig. 4d).

### YB-1 is critical for acidity-induced EndMT in HUVECs

Reports demonstrated that YB-1 was overexpressed in pancreatic cancer tissues and associated with potential metastasis and poor prognosis [28, 41]. Evdokimova et al. also identified that YB-1 induced EMT accompanied by the enhanced metastatic potential in breast cancer [27]. To further address the potential contribution of YB-1 to EndMT in HUVECs under acidic conditions, we first assessed the expression of YB-1 in the HUVECs with different pH values. As expected, the expression of YB-1 was increased both at mRNA and protein levels in acidity (pH 7.0, pH 6.8) (Additional file 2: Fig. S2a, f). To further explore the function of YB-1 in HUVECs under acidic conditions, small interfering RNA (siRNA) of YB-1 was applied. The silencing effect of si-YB-1 was confirmed at the mRNA and protein levels by qRT-PCR and western blot assay, respectively (Additional file 2: Fig. S2b, g). Results showed that knockdown of YB-1 significantly reversed acidity-induced EndMT in HUVECs which down-regulated the expression of α-SMA and Vimentin, but up-regulated the expression of VE-cadherin and CD31 (Additional files 2, 3: Figs. S2c, S3d). In addition, knockdown of YB-1 also restricted the motility, migration (Additional file 2: Fig. S2d, e), and permeability (Additional file 3: Fig. S3a) of HUVECs under acidic conditions. Furthermore, knockdown of YB-1 in HUVECs reduced the transendothelial migration of BxPC-3 and PANC-1 cells under acidic conditions (Additional file 3: Fig. S3b, c).

**Fig. 5:**
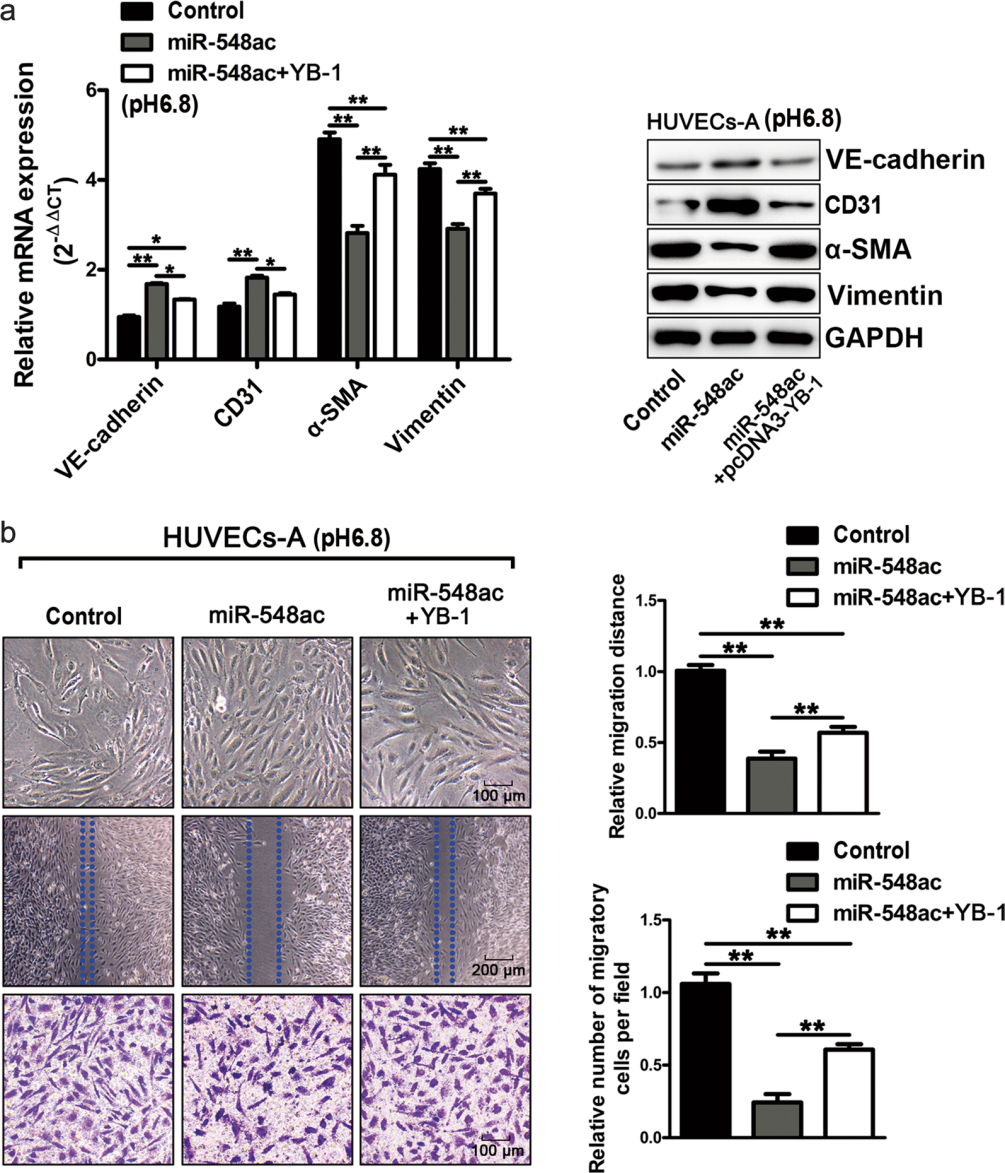
YB-1 is indispensable for miR-548ac deficiency-induced EndMT in HUVECs under acidic conditions. In the acidic medium (pH 6.8), after HUVECs were transfected with miR-548ac mimics alone or co-transfected with YB-1 overexpression plasmid. **a** The expression of VE-cadherin, CD31, α-SMA, and Vimentin were measured at mRNA and protein levels, respectively. **b** The morphology, wound-healing, and transwell migration assay of HUVECs were performed as indicated. Histograms are the statistical result of the corresponding wound-healing and transwell migration assay

To further validate the effect of the miR-548ac/YB-1 pathway on the acidity-induced EndMT, HUVECs were pretreated in pH6.8 medium and the acidity-treated HUVECs (HUVECs-A) were co-transfected with miR-548ac mimic and plasmid pcDNA3-YB-1 as well as their controls respectively. Compared to HUVECs-A transfected with miR-548ac mimic alone, HUVECs-A co-transfected with miR-548ac mimic and plasmid pcDNA3-YB-1 displayed significantly decreased expression of VE-cadherin and CD31 but increased expression of α-SMA and Vimentin both at mRNA and protein levels respectively (Fig. 5a; Additional file 3: Fig. S3e). Furthermore, the motility, permeability, and migration of co-transfected HUVECs-A were enhanced by wound-healing and transwell assay (Fig. 5b). In addition, our results also revealed that the restoration of YB-1 significantly impaired the inhibiting effect of miR-548ac on the permeability of HUVECs (Additional file 4: Fig. S4a) and the transendothelial migration of BxPC-3 and PANC-1 cells in the HUVECs barrier (Additional file 4: Fig. S4b, c).

Collectively, our data confirmed that YB-1 was a direct target of miR-548ac in HUVECs, which was responsible for the EndMT of HUVECs and enhanced the transmigration of pancreatic cancer cells.

### YB-1 induces EndMT of HUVECs by activating translation of Snail

**Fig. 6:**
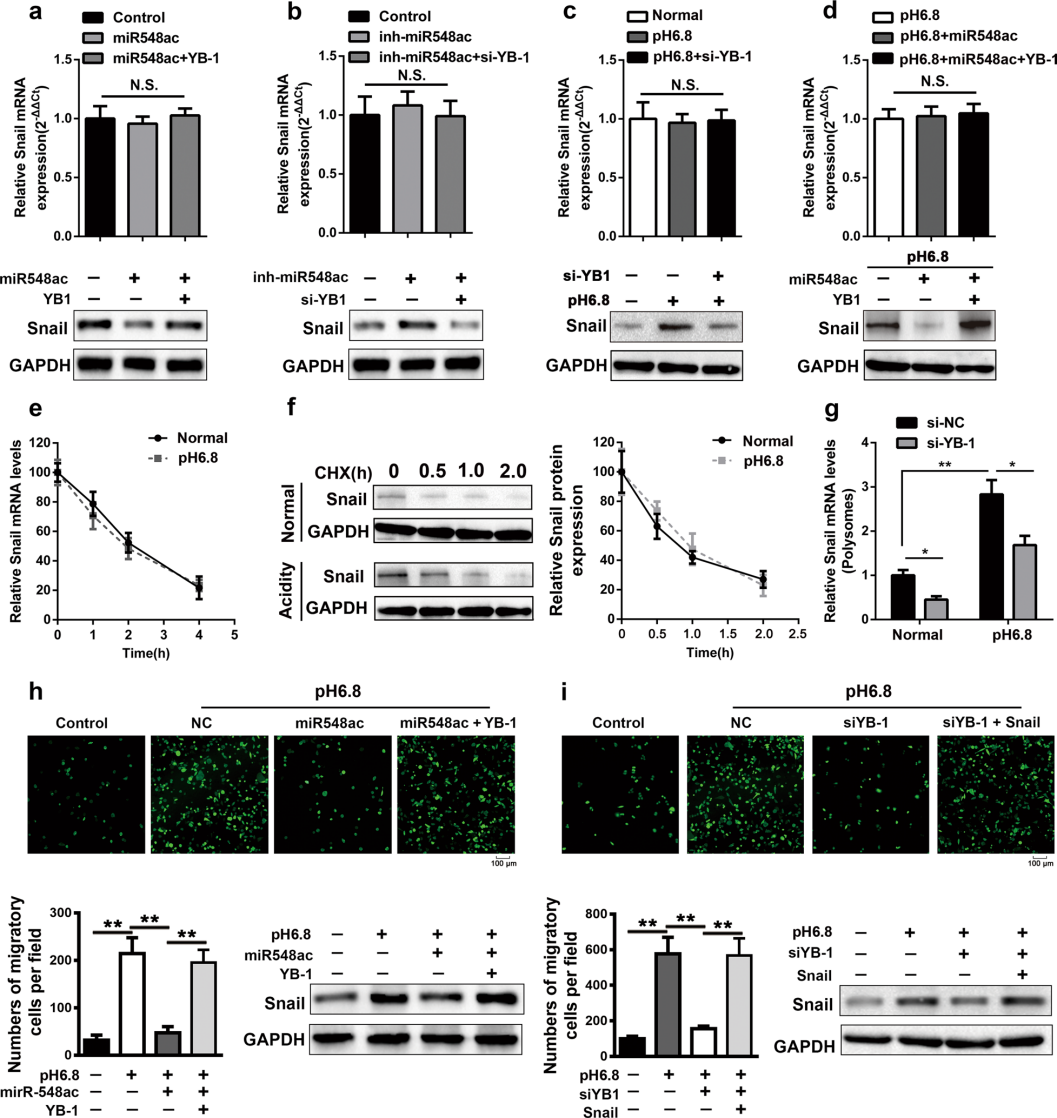
YB-1 activated Snail translation in HUVECs. **a** In the normal medium, after HUVECs were transfected with miR-548ac mimics alone or along with YB-1 overexpression plasmid, the expression of Snail mRNA and protein was measured by qPCR and western blot, respectively. **b** In the normal medium, after HUVECs were transfected with miR-548ac inhibitors alone or along with YB-1 siRNAs, the expression of Snail mRNA and protein was measured by qPCR and western blot, respectively. **c** The HUVECs cells were cultured in the normal, acidic medium or acidic medium transfected with YB-1 siRNAs. Then the Snail mRNA and protein level were measured by qPCR and western blot, respectively. **d** In an acidic medium, after HUVECs were transfected with miR-548ac mimics alone or along with a YB-1 overexpression plasmid, the expression of Snail mRNA and protein was measured by qPCR and western blot, respectively. **e** The HUVECs were cultured in the normal and acidic medium, and then both groups were treated with actinomycin D (1 μg/mL) as indicated time, the expression of Snail mRNA was measured by qPCR. **f** The HUVECs in the normal and acidic medium were both treated with CHX (10 μg/mL), and the level of Snail protein was measured by western blot. **g** The polysome analysis was performed in both normal and acidic mediums after YB-1 was knockdown in HUVECs. **h** The HUVECs permeability and transendothelial migration of BxPC-3 cells, as well as the level of Snail protein, were measured when HUVECs were divided into four groups, normal, acidity, acidity with miR-548ac mimics and acidity co-transfected with miR-548ac mimics, and YB-1 overexpression plasmid. **i** HUVECs permeability and transendothelial migration of BxPC-3 cells as well as the Snail protein level were measured when HUVECs were divided into four groups, normal, acidity, acidity with YB-1 siRNAs, and acidity co-transfected with YB-1 siRNAs and Snail overexpression plasmid

YB-1 plays important roles in almost mRNA and DNA dependent processes including DNA replication and repair, transcription, and mRNA translation [42], which is involved in malignant transformation, cell invasion, and drug resistance in certain cancers [27, 43]. Since YB-1 has been known as a critical regulator that directly activated cap-independent translation of messenger RNA encoding Snail, we further assessed whether YB-1 contributed to the regulation of Snail in HUVECs under the acidic microenvironment. Our results showed that overexpression of YB-1 could abolish the inhibitory effect of miR-548ac on Snail protein expression without affecting its mRNA level in HUVECs. In turn, the elevation of Snail protein induced by inhibition of miR-548ac was inhibited when YB-1 was knockdown, also without significant change at the mRNA level of Snail (Fig. 6a, b; Additional file 3: Fig. S3f, g). Simultaneously, the acidity-induced elevation of Snail protein was inhibited by YB-1 knockdown, while overexpression of YB-1 could also rescue Snail protein from the inhibitory effect of miR-548ac overexpressed in the acidic environment, and there was no significant change at mRNA level (Fig. 6c, d; Additional file 3: Fig. S3h, S4d). The results demonstrated that miR-548ac might suppress Snail protein expression through inhibition of YB-1. Further, Snail was reported to be a direct target of HIF-1α in hypoxia-induced EndMT [44], and it was reported that YB-1 could improve the translational efficiency of Snail mRNA [27]. Our study also showed the acidic microenvironment did not affect the stability of Snail mRNA and protein (Fig. 6e, f). However, the polysome-bound Snail mRNA level was increased in the acidic environment, and knockdown of YB-1 protein reduced the polysome-bound level of Snail mRNA (Fig. 6g), indicating YB-1 protein promoted the translation of Snail mRNA. Moreover, the acidity-induced enhancement of HUVECs permeability and transendothelial migration of BxPC-3 cells could be repressed by overexpression of miR-548ac or knockdown of YB-1 coupled with Snail protein downregulation, while overexpression of YB-1/Snail could offset the inhibitory effects, simultaneously accompanied with upregulation of Snail protein (Fig. 6h, i; Additional file 4: Fig. S4e, f).

These results indicated that the transcriptional factor Snail was translationally upregulated by YB-1, which was importantly required in the EndMT of HUVECs during acidity.

### Snail reciprocally inhibits the transcription of miR-548ac under acidic condition

**Fig. 7:**
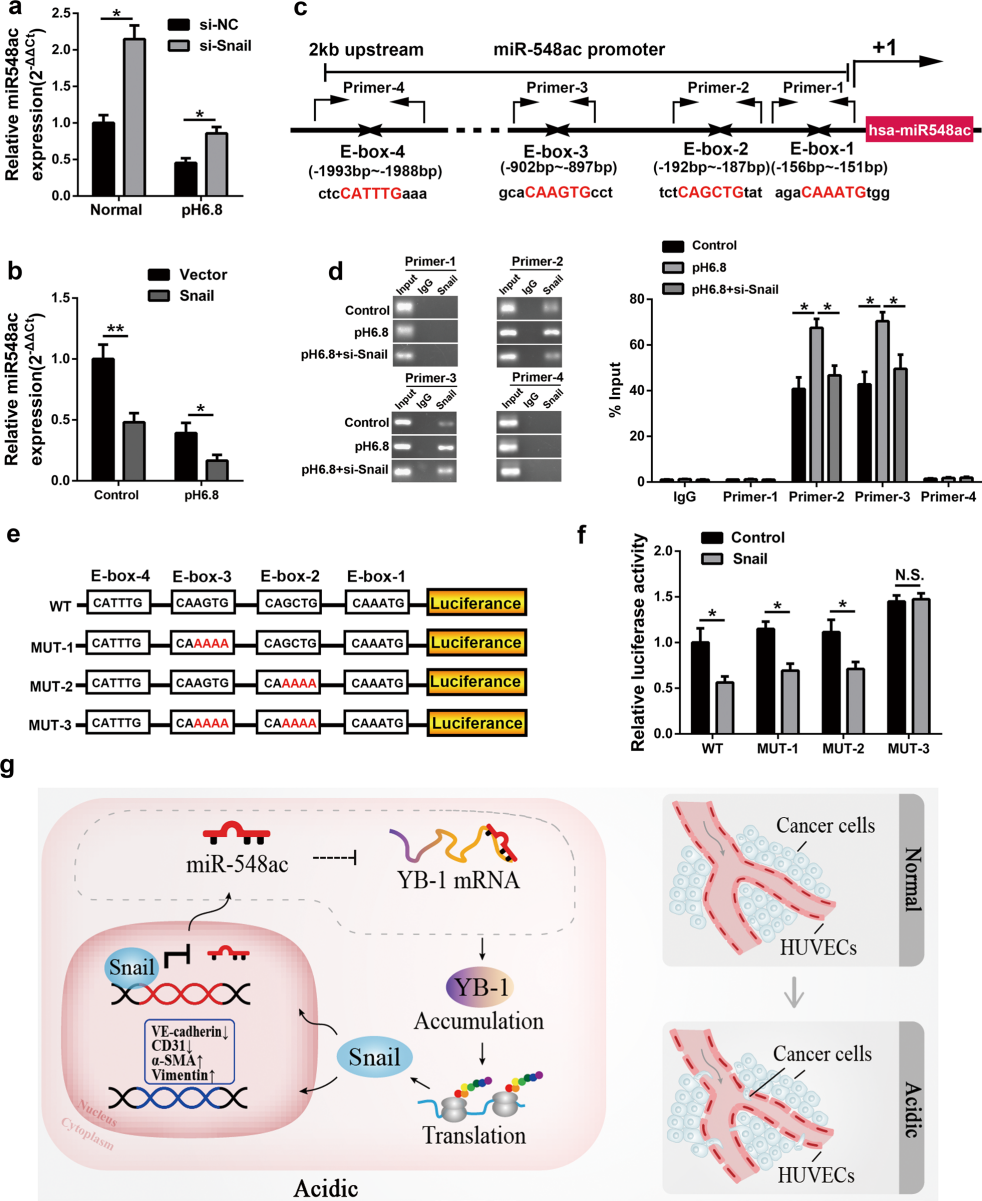
Snail inhibits transcription of miR-548ac under acidic conditions. **a**, **b** The expression of miR-548ac was measured by qPCR when HUVECs were transfected with Snail siRNAs or overexpression plasmid in the normal or acidic medium. **c** The schematic shows that the host-gene of miR-548ac harbors four E-box elements (E-box-1, E-box-2, E-box-3, E-box-4) that may be combined by Snail protein. **d** The ChIP analysis of the binding capacity between Snail protein and the E-box sequences on the promoter of miR-548ac host-gene in the Control, Acidity, and Acidity with Snail knockdown groups, respectively. **e** We constructed four luciferase reporter plasmids to identify the promoter transcriptional activity of miR-548ac host-gene, wild type (WT) without any mutation, MUT-1 with E-box-3 mutation, MUT-2 with E-box-2 mutation, and MUT3 with both mutations of E-box-2 and E-box-3. **f** The luciferase activity of WT, MUT-1, MUT-2, MUT-3 plasmids co-transfected with Snail overexpression plasmid or control vector. **g** The schematic summary shows that in the acidic environment-induced low expression of miR-548ac mediated the YB-1/Snail positive feedback pathway leads to the metastasis of pancreatic cancer cells by increasing the permeability of HUVECs

Since the data demonstrated a direct regulatory linkage among the single pathway of miR-548ac/YB-1/Snail. Snail is an inhibitory transcription factor [45] that promotes endothelial to mesenchymal transition in HUVECs [46]. We further determined whether Snail could regulate the expression of miR-548ac directly. As predicted, our findings revealed that knockdown of Snail impaired the downregulation of miR-548ac in the acidity-treated HUVECs, while overexpression of Snail led to a strong reduction in the expression of miR-548ac (Fig. 7a, b).

Recent studies demonstrated that the upstream DNA sequence of the miRNA host gene might function as a promoter of miRNA, which displayed the capacity of transcriptional regulation [47, 48]. To study the transcriptional regulation, we isolated the 2 kb upstream region of the miR-548ac host gene promoter and analyzed the binding capacity for various transcription factors. Intriguingly, the sequence analysis identified four potential consensus motifs (E-box-1–E-box-4) for Snail binding within the − 151 and − 1993 regions from the host gene transcription initiation site of miR-548ac (Fig. 7c). To validate the binding capacity of Snail on miR-548ac, the chromatin immunoprecipitation (ChIP) assay was applied to verify the putative Snail binding sites. The ChIP assay showed that Snail was recruited to the regions encompassing − 187 to − 192 (E-box-2) and − 897 to − 902 (E-box-3), which contained an E-box site (CAGCTG/CAAGTG) respectively, and these binding were enhanced in acidity (Fig. 7d). Additionally, to validate the function of Snail binding to the promoter of miR-548ac, four luciferase reporter plasmids containing the promoter of miR-548ac described in Fig. 7e were transfected into HUVECs, respectively, along with pRL-TK as internal reference plasmid. The results of the luciferase assay showed both E-box-2 and E-box-3 were functional Snail binding sites, and luciferase activity in transfected HUVECs was significantly decreased when overexpression of Snail (Fig. 7f). These data suggested that miR-548ac could be transcriptionally inhibited by Snail under acidic conditions. Figure 7g was a schematic diagram of the mechanism involved herein. We revealed that miR-548ac regulated the expression of Snail in a YB-1-dependent manner, and Snail inhibited the transcription of the miR-548ac host gene.

Taken together, our research identified a functional positive feedback loop of the miR-548ac/YB-1/Snail axis, which could be the trigger under acidic microenvironment, contributing to the endothelial to mesenchymal transition of HUVECs, further led to a significant increase of endothelial cell permeability, consequently facilitated the malignant progression of pancreatic cancer.

## Discussion

Endothelial cells (ECs) are the building blocks of the vascular system, forming the inner lining of blood vessels and lymphatics. Vascular ECs were initially considered to play an active role in regulating cardiovascular and systemic homeostasis by gating the traffic of molecules and cells across the vessel wall. Recently, increasing evidence revealed that ECs demonstrated functional and phenotypic heterogeneity when located under different microenvironments. A characteristic example of such phenotypic modification is EndMT, which is implicated in the progression of tumor through complex modulation of the tumor and its microenvironment, during which ECs lose their endothelial phenotype and acquire mesenchymal traits with an increase of endothelial permeability. Several experiments highlighted the critical role of vascular hyperpermeability in the extravasation of tumor cells. In addition, it is now accepted the stimuli in the tumor microenvironment can cause endothelial barrier dysfunction, however, the molecular mechanisms involved in are still poorly understood. In our present study, we described a tumor microenvironment-stimulated EndMT of ECs and investigated its impact on migration and metastasis of pancreatic cancer.

The major finding of our present study was that the acidic microenvironment could promote the acquisition of EndMT in ECs, thereby contributing to the vascular hyperpermeability and extravasation of pancreatic cancer cells. Currently, it is now accepted that ECs demonstrate a high degree of plasticity under different microenvironment stimuli, which facilitate tumor development. Strikingly, many different endothelial cell types have demonstrated EndMT when exposed to tumor microenvironment stimuli. Xu et al. reported primary Human Coronary Endothelial Cells underwent EndMT in response to hypoxia via HIF1α-mediated induction of Snail [44]. Park et al. suggested hypoxia/aglycemia decreased microvascular endothelial permeability through PKC, PKG, MAP kinase, and Ca^2+^ that was related to the dissociation of cadherin-actin and occludin-actin junctional bonds [49]. Endothelial cells are heavily exposed to Reactive Oxygen Species (ROS), which are involved in the signaling pathway regulating vascular permeability [50–53]. Studies also reported the loss of endothelial cell integrity and selective permeability barrier was an early event in the sequence of oxidant-mediated injury and might result in atherosclerosis, hypertension, and facilitation of transendothelial migration of cancer cells (TEM) during metastasis [35, 54–57]. In consist with that, our result showed that acidic pHe induced typical EndMT phenotypic changes in cultured HUVECs. Furthermore, in response to acidity, HUVECs monolayers demonstrated a profound increase of endothelial permeability, facilitating the transendothelial migration of pancreatic cancer cells (BxPC-3, PANC-1). In this regard, our study demonstrated that EndMT in HUVECs induced vascular hyperpermeability in the acidic microenvironment.

Another major accomplishment of our present study was further delineating one of the pathways which explained how acidity triggered EndMT and consequently facilitated the malignant progression of pancreatic cancer. Recently, a wealth of evidence from our laboratory and others highlighted the role of miRNAs in the EMT process [20–22, 24]. Importantly, Ghosh et al. found the expression levels of specific miRNAs, known to be dysregulated in different cardiovascular diseases, were altered during EndMT by a microRNA array [25]. Zhu et al. also reported miR-302c inhibited the growth of hepatocellular carcinoma by suppressing the EndMT of endothelial cells (ECs) [26]. Studies on pancreatic cancer also showed a decisive role of microRNAs in the regulation of tumor invasion and metastasis. For pancreatic cancer, acidity is a hallmark in the tumor microenvironment and a principal inducer of aggressive biological behaviors of pancreatic cancer cells [20, 58]. Recent reports emphasized microRNAs acted as a stress-responsive mediator in signal transduction facilitating the interaction between tumor cells and the tumor microenvironment in response to stress including hypoxia, inflammation, and acidity [20–23]. In our study, we applied the miRNAs microarray to explore the miRNAs panel involved in the acidity-induced EndMT of HUVECs. Among those dysregulating miRNAs in acidity, miR-548ac was an attractive candidate with significant downregulation in the acidic microenvironment. Further, we also observed that acidic treatment increased the expression of YB-1 and Snail in a pH dependent manner, combined with an EndMT-like transformation of HUVECs, leading to an increase of endothelial permeability and facilitation of transendothelial migration of pancreatic cancer cells (BxPC-3, PANC-1).

Further, functional studies in vitro revealed overexpression of miR-548ac elicited the opposite effect as described above, demonstrating the suppressive role of miR-548ac in acidity-induced EndMT of HUVECs, which was comparable with the previous studies [26]. Recently, numerous studies pointed out the role of YB-1 in the progression of tumor. However, the exact role of YB-1 in this process was ambiguous since it had been described as both a tumor suppressor and an oncogene [59, 60]. YB-1 revealed its pro-tumorigenic activity as a transcriptional activator in the nucleus-induced proliferation by activating pro-growth genes through binding to the Y-box elements in their promoter region. In contrast, in the cytoplasm, YB-1 could possess tumor suppressor properties by silencing translation through binding to the 5′ terminus of mRNAs of genes related to the growth and proliferation of tumor cells [60]. In our present study, consistent with the observed EndMT in acidity-treated HUVECs, the expression of YB-1 was increased in a pH dependent manner and was inversely correlated with the expression of miR-548ac. More importantly, our study showed that miR-548ac inhibited the expression of YB-1. Bioinformatics analysis and luciferase reporter assays demonstrated YB-1 was a direct target of miR-548ac. Further, functional studies showed that knockdown of YB-1 significantly reversed acidity-induced EndMT of HUVECs, while ectopic restoration of YB-1 significantly impaired the suppressive effect of miR-548ac on EndMT of HUVECs treated in acidity as well as the permeability and the transendothelial migration of BxPC-3 and PANC-1 cells through HUVECs monolayer. In this regard, our findings were in line with existing literature, demonstrating the prooncogenic role of YB-1.

Interestingly, YB-1 was identified as a crucial factor in the regulation of EMT through a novel mechanism, involving the translation of cap-independent mRNAs including Snail1 and Twist [27]. Hence, our present study assessed whether YB-1 contributed to the regulation of Snail in HUVECs under the acidic microenvironment. Our present results further confirmed that YB-1 enhanced Snail protein expression by activating the translation of Snail. Consistently, ectopic expression of YB-1 led to the induction of α-SMA and Vimentin, reduction of VE-cadherin and Zo-1 both at mRNA and protein levels and enhanced the motility, permeability of HUVECs as well as transendothelial migration of BxPC-3 and PANC-1 cells. Evdokimova et al. suggested translational regulation by YB-1 was a restriction point during metastatic progression while facilitating activation of mesenchymal phenotype. The process of mesenchymal transformation appeared to be a two-step mechanism involving Ras-ERK-dependent transcription of Snail and other developmentally regulated genes and YB-1-mediated translation of the transcripts into protein products. In turn, these new protein products, such as Snail, were responsible for subsequent transcriptional repression of E-cadherin and other epithelial genes and activation of mesenchymal genes [27]. In this regard, our results confirmed that YB-1 induced translational activation of Snail, and Snail mediated the EndMT of HUVECs.

Recent studies demonstrated that the upstream DNA sequence of miRNA might function as a promoter of miRNA, which displayed the capacity of transcriptional regulation [47, 48]. Our previous study also demonstrated miR-548an could be transcriptionally downregulated by HIF1α/HDAC1, further suppressing tumorigenesis of pancreatic cancer [61]. Snail has been regarded as a universal transcriptional repressor and involved in cancer regulation through an E-box sequence with a consensus of six base pairs (CANNTG) [33]. In line with that, we revealed that Snail could directly bind to two E-box motifs within the upstream 2 kb region of the miR-548ac promoter and repressed its expression. In this regard our results demonstrated that miR-548ac/YB-1/Snail signaling pathway formed a dynamic feedback loop.

## Conclusions

In summary, our present study identified a biochemical function link among acidic microenvironment, miR-548ac, YB-1, and Snail in HUVECs. Further, the repression of miR-548ac resulted in the YB-1/Snail axis forming a feedback loop, which helped to regulate the function of ECs. In our study, this feedback loop demonstrated a positive effect in response to the acidic microenvironment, where the expression of miR-548ac was repressed in HUVECs treated with acidity, which attenuated the direct suppression of miR-548ac on YB-1. Consequently, augmented the translational activation of Snail by YB-1, and, in turn, Snail repressed the expression of miR-548ac by binding to the promoter of the host gene and inhibiting its transcription. This dynamic process further led to a significant increase in endothelial cell permeability, facilitating the malignant progression of pancreatic cancer. In addition, our results provided compelling evidence for further rationalizing the targeting strategy in clinical practice to treat pancreatic cancer.

## Supplementary Information

**Additional file 1: Figure S1.** MiR-548ac overexpression significantly receded the transendothelial migration of pancreatic cancer cells from HUVECs barrier. **a** The passage of Rhodamine-labelled dextran was measured to analyze the permeability of HUVECs under condition of pH = 7.4, pH = 6.8, or pH = 6.8 with miR-548ac mimics. **b**, **c** The transendothelial migration assay of BxPC-3 and PANC-1 cells crossed the HUVECs monolayers on pH = 7.4, pH = 6.8, or pH = 6.8 with miR-548ac mimics. The histogram represents the relative migrated number of BxPC-3 and PANC-1 cells. **d** The viability of HUVECs cells were measured on the pH = 7.4, pH = 7.0, or pH = 6.8 conditions. **e** The expression of vascular endothelial markers including VE-cadherin, CD31, α-SMA, and Vimentin at protein levels in normal and conditioned medium (pH 7.0 pH 6.8). **f** The expression of vascular endothelial markers including VE-cadherin, CD31, α-SMA, and Vimentin at protein levels in HUVECs in normal, acidic medium or acidic medium with miR-548ac mimics. **g** After inhibition or overexpression of miR-548ac in the normal or acidic medium, the expression of YB-1 mRNA and protein were measured by western blot. All data were revealed as means ± standard deviation (SD) for no less than three independent experiments. Significant P values showed as *P < 0.05 and **P < 0.01.

**Additional file 2: Figure S2.** YB-1 promoted EndMT in HUVECs under acidic condition. **a** The expression of YB-1 mRNA and protein level in HUVECs under pH = 7.4 and pH = 6.8 condition was measured by qPCR and western blot, respectively. **b** The knockdown efficiency of siRNAs targeting YB-1 was measured by qPCR and western blot, respectively. **c** The expression of vascular endothelial markers including VE-cadherin, CD31, α-SMA and Vimentin at mRNA and protein levels in HUVECs in pH = 7.4, pH = 6.8 medium or pH = 6.8 medium with YB-1 knockdown. **d**, **e** The morphology and migration of HUVECs as well as the transendothelial migration of BxPC-3 were analysed, respectively. **f** The expression of YB-1 at protein level in HUVECs under pH = 7.4 and pH = 6.8 condition was measured by western blot. **g** The knockdown efficiency of siRNAs targeting YB-1 was measured western blot. The histogram represents relative migrated number of BxPC-3 cells. All data were revealed as means ± standard deviation (SD) for no less than three independent experiments. Significant P values showed as *P < 0.05 and **P < 0.01.

**Additional file 3: Figure S3.** Knockdown of YB-1 increased the permeability of HUVECs under acidic condition. **a** The passage of Rhodamine-labelled dextran was measured to analyze the permeability of HUVECs under condition of pH = 7.4, pH = 6.8, or pH = 6.8 with YB-1 knockdown. **b**, **c** The transendothelial migration assay of BxPC-3 and PANC-1 cells crossed the HUVECs monolayers in pH = 7.4 pH = 6.8 or pH = 6.8 with YB-1 knockdown conditions. **d** The expression of vascular endothelial markers including VE-cadherin, CD31, α-SMA and Vimentin at protein levels in HUVECs in pH = 7.4, pH = 6.8 medium or pH = 6.8 medium with YB-1 knockdown. **e** The expression of VE-cadherin, CD31, α-SMA, and Vimentin were measured at protein levels. **f** In the normal medium, after HUVECs were transfected with miR-548ac mimics alone or along with YB-1 overexpression plasmid, the expression of Snail protein was measured and western blot. **g** In the normal medium, after HUVECs were transfected with miR-548ac inhibitors alone or along with YB-1 siRNAs, the expression of Snail protein was measured by western blot. **h** The HUVECs cells were cultured in the normal, acidic medium or acidic medium transfected with YB-1 siRNAs. Then the Snail protein level were measured by western blot. The histogram represents relative migrated number of BxPC-3 and PANC-1 cells. All data were revealed as means ± standard deviation (SD) for no less than three independent experiments. Significant P values showed as *P < 0.05 and **P < 0.01.

**Additional file 4: Figure S4.** Overexpression of YB-1 impaired the inhibiting effect of miR-548ac on the permeability of HUVECs. **a** The passage of Rhodamine-labelled dextran was measured to analyze the permeability of HUVECs in normal, transfected with miR-548ac mimics alone or co-transfected with miR-548ac mimics and YB-1 overexpression plasmid. **b**, **c** The transendothelial migration assay of BxPC-3 and PANC-1 cells crossed the HUVECs monolayers in in normal, transfected with miR-548ac mimics alone or co-transfected with miR-548ac mimics and YB-1 overexpression plasmid. **d** In an acidic medium, after HUVECs were transfected with miR-548ac mimics alone or along with a YB-1 overexpression plasmid, the expression of Snail protein was measured by western blot. **e** The level of Snail protein, were measured when HUVECs were divided into four groups, normal, acidity, acidity with miR-548ac mimics and acidity co-transfected with miR-548ac mimics, and YB-1 overexpression plasmid. **f** The Snail protein level were measured when HUVECs were divided into four groups, normal, acidity, acidity with YB-1 siRNAs, and acidity co-transfected with YB-1 siRNAs and Snail overexpression plasmid. The histogram represents relative migrated number of BxPC-3 and PANC-1 cells. All data were revealed as means ± standard deviation (SD) for no less than three independent experiments. Significant P values showed as *P < 0.05 and **P < 0.01. N.S. means the difference was not significant.

**Additional file 5: Table S1.** The sequence of primers.

## Data Availability

The data supporting the conclusions of this article are included within the article and its additional files. Further details are available from the corresponding author on request.

## Abbreviations

EndMT: Endothelial-to-mesenchymal transition
ECs: Endothelial cells
pHe: Extracellular pH
ROS: Reactive oxygen species
TEM: Transendothelial migration
HUVECs: Human umbilical vein endothelial cells
YB-1: Y-box binding protein-1
WT: Wild-type
MUT: Mutant-type
