# Supplementary figures and images for: A reciprocal feedback of miR-548ac/YB-1/Snail induces EndMT of HUVECs during acidity microenvironment
Source: Cancer Cell Int. 2021 Dec 20;21:692. doi: 10.1186/s12935-021-02388-8 (PMC8691019; doi:10.1186/s12935-021-02388-8)

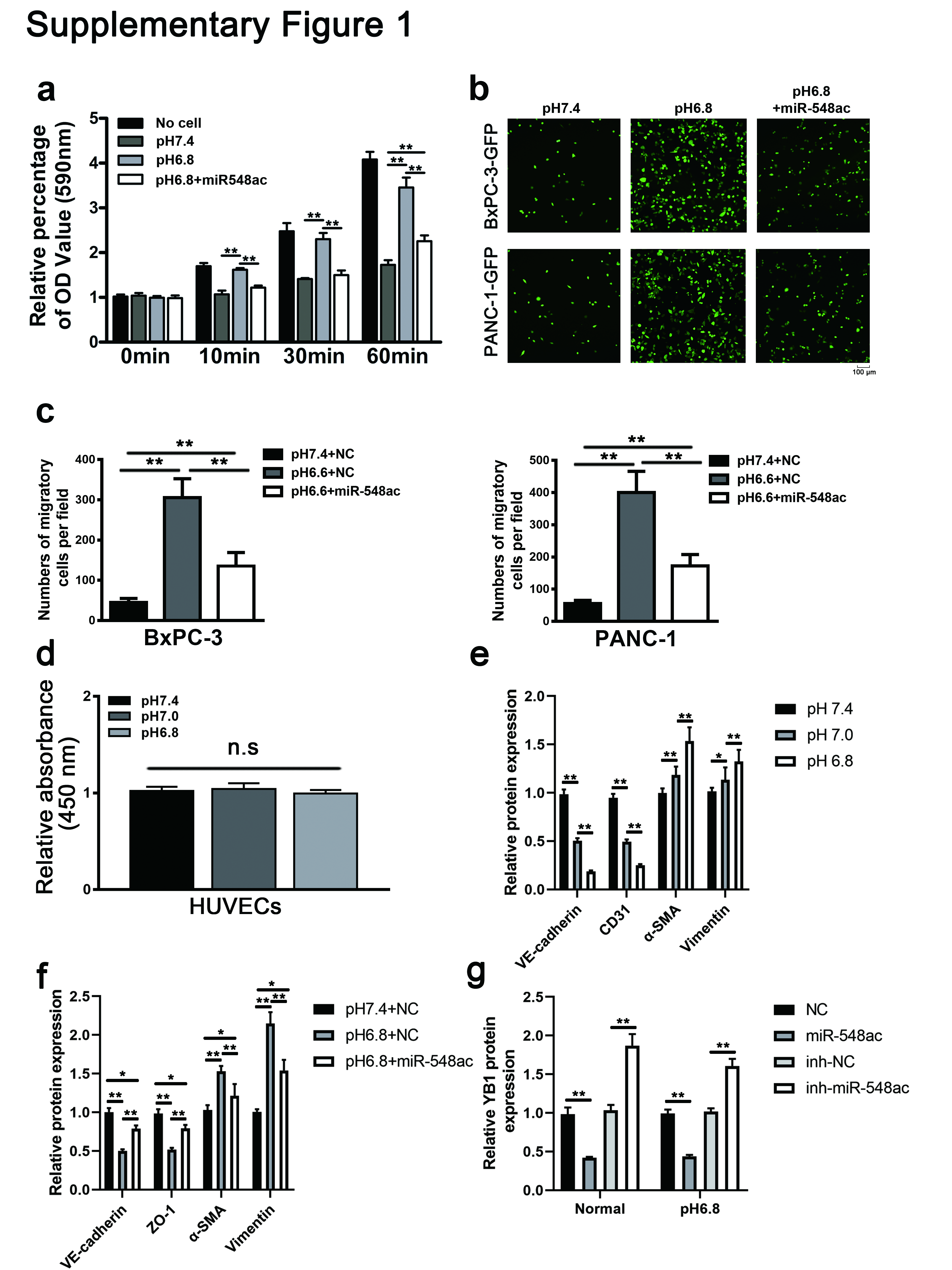

Supplement: Supplementary file 1 — Additional file 1: Figure S1. MiR-548ac overexpression significantly receded the transendothelial migration of pancreatic cancer cells from HUVECs barrier. a The passage of Rhodamine-labelled dextran was measured to analyze the permeability of HUVECs under condition of pH = 7.4, pH = 6.8, or pH = 6.8 with miR-548ac mimics. b, c The transendothelial migration assay of BxPC-3 and PANC-1 cells crossed the HUVECs monolayers on pH = 7.4, pH = 6.8, or pH = 6.8 with miR-548ac mimics. The histogram represents the relative migrated number of BxPC-3 and PANC-1 cells. d The viability of HUVECs cells were measured on the pH = 7.4, pH = 7.0, or pH = 6.8 conditions. e The expression of vascular endothelial markers including VE-cadherin, CD31, α-SMA, and Vimentin at protein levels in normal and conditioned medium (pH 7.0 pH 6.8). f The expression of vascular endothelial markers including VE-cadherin, CD31, α-SMA, and Vimentin at protein levels in HUVECs in normal, acidic medium or acidic medium with miR-548ac mimics. g After inhibition or overexpression of miR-548ac in the normal or acidic medium, the expression of YB-1 mRNA and protein were measured by western blot. All data were revealed as means ± standard deviation (SD) for no less than three independent experiments. Significant P values showed as *P < 0.05 and **P < 0.01. [file 12935_2021_2388_MOESM1_ESM.tif]

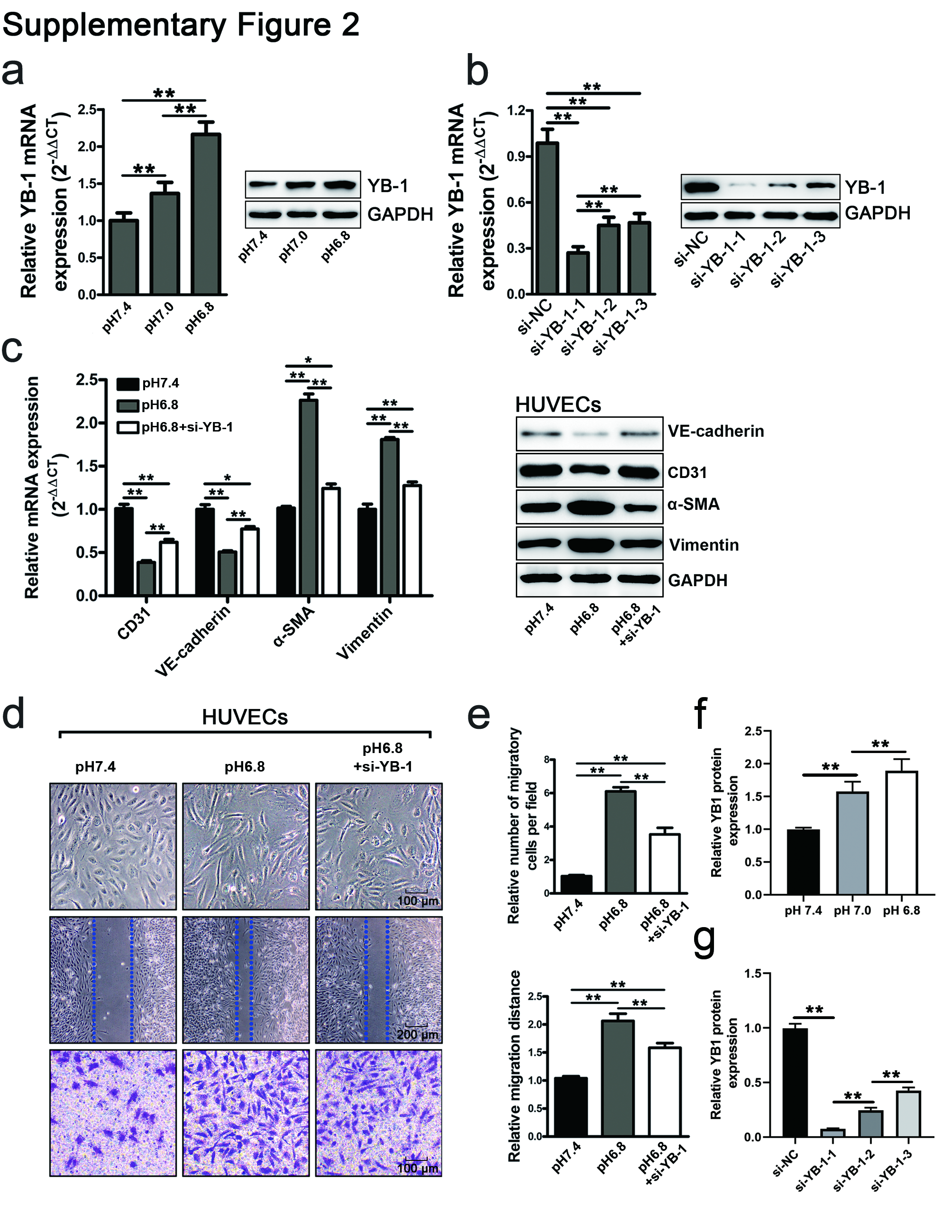

Supplement: Supplementary file 2 — Additional file 2: Figure S2. YB-1 promoted EndMT in HUVECs under acidic condition. a The expression of YB-1 mRNA and protein level in HUVECs under pH = 7.4 and pH = 6.8 condition was measured by qPCR and western blot, respectively. b The knockdown efficiency of siRNAs targeting YB-1 was measured by qPCR and western blot, respectively. c The expression of vascular endothelial markers including VE-cadherin, CD31, α-SMA and Vimentin at mRNA and protein levels in HUVECs in pH = 7.4, pH = 6.8 medium or pH = 6.8 medium with YB-1 knockdown. d, e The morphology and migration of HUVECs as well as the transendothelial migration of BxPC-3 were analysed, respectively. f The expression of YB-1 at protein level in HUVECs under pH = 7.4 and pH = 6.8 condition was measured by western blot. g The knockdown efficiency of siRNAs targeting YB-1 was measured western blot. The histogram represents relative migrated number of BxPC-3 cells. All data were revealed as means ± standard deviation (SD) for no less than three independent experiments. Significant P values showed as *P < 0.05 and **P < 0.01. [file 12935_2021_2388_MOESM2_ESM.tif]

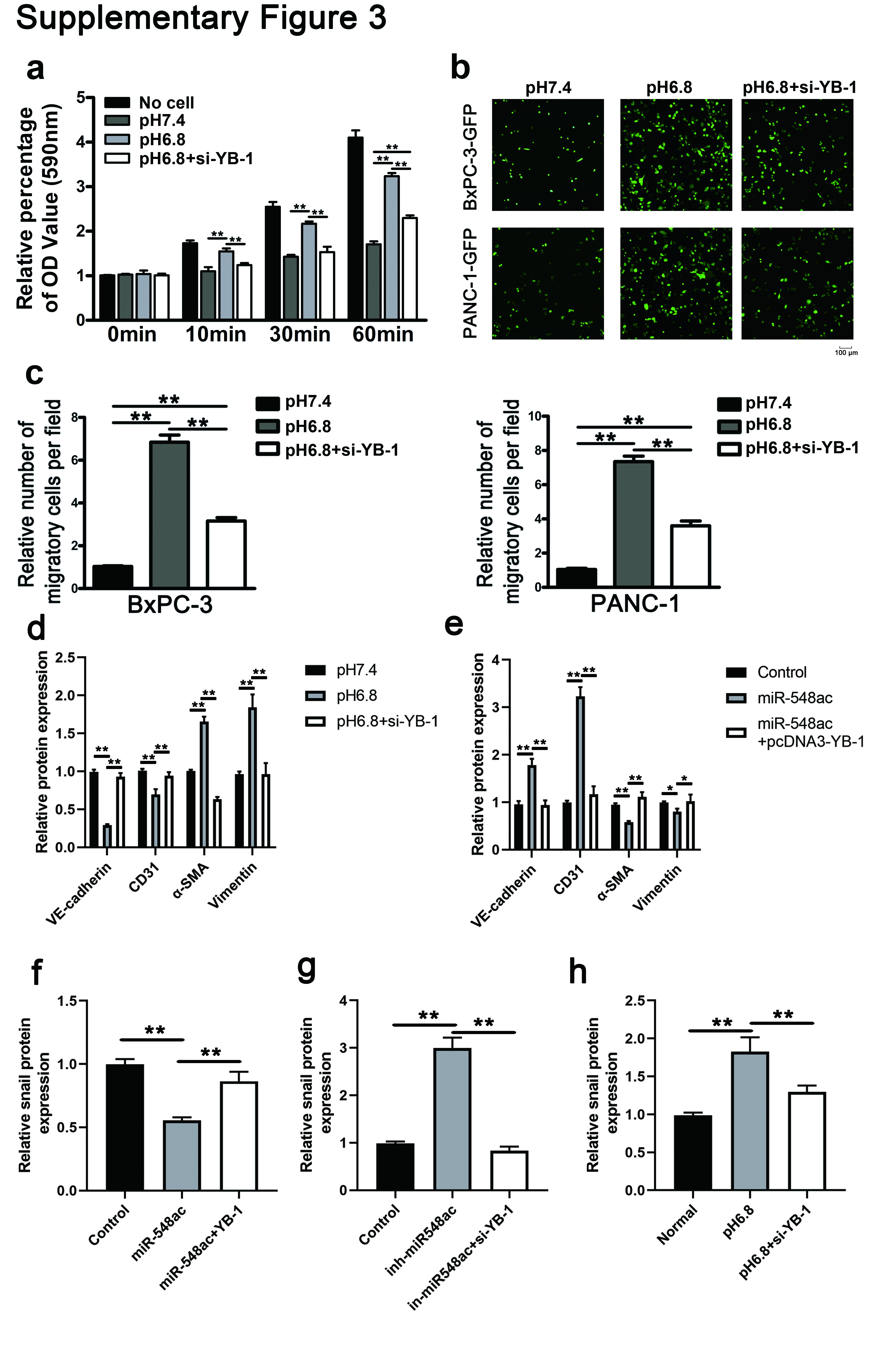

Supplement: Supplementary file 3 — Additional file 3: Figure S3. Knockdown of YB-1 increased the permeability of HUVECs under acidic condition. a The passage of Rhodamine-labelled dextran was measured to analyze the permeability of HUVECs under condition of pH = 7.4, pH = 6.8, or pH = 6.8 with YB-1 knockdown. b, c The transendothelial migration assay of BxPC-3 and PANC-1 cells crossed the HUVECs monolayers in pH = 7.4 pH = 6.8 or pH = 6.8 with YB-1 knockdown conditions. d The expression of vascular endothelial markers including VE-cadherin, CD31, α-SMA and Vimentin at protein levels in HUVECs in pH = 7.4, pH = 6.8 medium or pH = 6.8 medium with YB-1 knockdown. e The expression of VE-cadherin, CD31, α-SMA, and Vimentin were measured at protein levels. f In the normal medium, after HUVECs were transfected with miR-548ac mimics alone or along with YB-1 overexpression plasmid, the expression of Snail protein was measured and western blot. g In the normal medium, after HUVECs were transfected with miR-548ac inhibitors alone or along with YB-1 siRNAs, the expression of Snail protein was measured by western blot. h The HUVECs cells were cultured in the normal, acidic medium or acidic medium transfected with YB-1 siRNAs. Then the Snail protein level were measured by western blot. The histogram represents relative migrated number of BxPC-3 and PANC-1 cells. All data were revealed as means ± standard deviation (SD) for no less than three independent experiments. Significant P values showed as *P < 0.05 and **P < 0.01. [file 12935_2021_2388_MOESM3_ESM.tif]

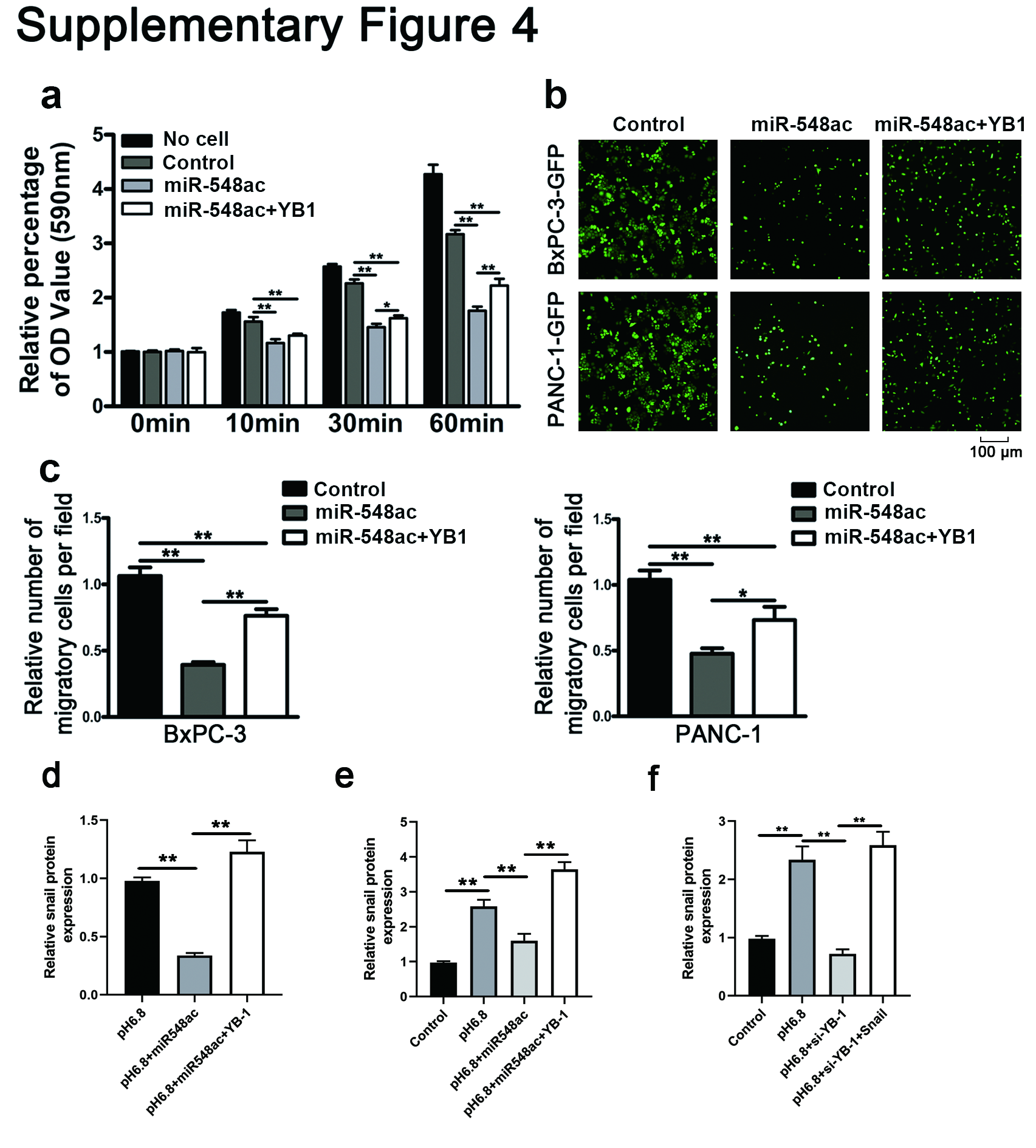

Supplement: Supplementary file 4 — Additional file 4: Figure S4. Overexpression of YB-1 impaired the inhibiting effect of miR-548ac on the permeability of HUVECs. a The passage of Rhodamine-labelled dextran was measured to analyze the permeability of HUVECs in normal, transfected with miR-548ac mimics alone or co-transfected with miR-548ac mimics and YB-1 overexpression plasmid. b, c The transendothelial migration assay of BxPC-3 and PANC-1 cells crossed the HUVECs monolayers in in normal, transfected with miR-548ac mimics alone or co-transfected with miR-548ac mimics and YB-1 overexpression plasmid. d In an acidic medium, after HUVECs were transfected with miR-548ac mimics alone or along with a YB-1 overexpression plasmid, the expression of Snail protein was measured by western blot. e The level of Snail protein, were measured when HUVECs were divided into four groups, normal, acidity, acidity with miR-548ac mimics and acidity co-transfected with miR-548ac mimics, and YB-1 overexpression plasmid. f The Snail protein level were measured when HUVECs were divided into four groups, normal, acidity, acidity with YB-1 siRNAs, and acidity co-transfected with YB-1 siRNAs and Snail overexpression plasmid. The histogram represents relative migrated number of BxPC-3 and PANC-1 cells. All data were revealed as means ± standard deviation (SD) for no less than three independent experiments. Significant P values showed as *P < 0.05 and **P < 0.01. N.S. means the difference was not significant. [file 12935_2021_2388_MOESM4_ESM.tif]
