# Supplementary material for: A reciprocal feedback of miR-548ac/YB-1/Snail induces EndMT of HUVECs during acidity microenvironment
Source: Cancer Cell Int. 2021 Dec 20;21:692. doi: 10.1186/s12935-021-02388-8 (PMC8691019; doi:10.1186/s12935-021-02388-8)
Supplement: Supplementary file 5 — Additional file 5: Table S1. The sequence of primers. [file 12935_2021_2388_MOESM5_ESM.docx]

**Table S1.** The sequence of primers.

| Primer | Sequence |
| --- | --- |
| VE-cadherin | Forward: 5’-TTGGAACCAGATGCACATTGAT-3’ |
|  | Reverse: 5’-TCTTGCGACTCACGCTTGAC-3’ |
| CD31 | Forward: 5’-AACAGTGTTGACATGAAGAGCC-3’ |
|  | Reverse: 5’-TGTAAAACAGCACGTCATCCTT-3’ |
| α-SMA | Forward: 5’-CCTGTGTTGTGGTTTACACTGG-3’ |
|  | Reverse: 5’-GGGGGAATTATCTTTCCTGGTCC-3’ |
| Vimentin | Forward: 5’-GACGCCATCAACACCGAGTT-3’ |
|  | Reverse: 5’-CTTTGTCGTTGGTTAGCTGGT-3’ |
| YB-1 | Forward: 5’-GGGGACAAGAAGGTCATCGC-3’ |
|  | Reverse: 5’-CGAAGGTACTTCCTGGGGTTA-3’ |
| Snail | Forward: 5’-TCGGAAGCCTAACTACAGCGA-3’ |
|  | Reverse: 5’-AGATGAGCATTGGCAGCGAG-3’ |
| GAPDH | Forward: 5’-GGAGCGAGATCCCTCCAAAAT-3’ |
|  | Reverse: 5’-GGCTGTTGTCATACTTCTCATGG-3’ |
